# Crossmodal plasticity and hearing capabilities following blindness

**DOI:** 10.1007/s00441-015-2175-y

**Published:** 2015-04-18

**Authors:** Andrew J. King

**Affiliations:** Department of Physiology, Anatomy and Genetics, University of Oxford, Oxford, UK

**Keywords:** Blindness, Auditory localization, Cortex, Experience-dependent plasticity, Functional reorganization

## Abstract

Valuable insights into the role of experience in shaping perception can be obtained by studying the effects of blindness or other forms of sensory deprivation on the intact senses. Blind individuals are particularly dependent on their hearing and there is extensive evidence that they can develop superior auditory skills, either as a result of plasticity within the auditory system or through the recruitment of functionally relevant occipital cortical areas that lack their normal visual inputs. Because spatial processing normally relies on close interactions between vision and hearing, much of the research in this area has focused on the effects of blindness on auditory localization. Although enhanced auditory skills have been reported in many studies, some aspects of spatial hearing are impaired in the absence of vision. In this case, the effects of crossmodal plasticity may reflect a balance between adaptive changes that compensate for blindness and the role vision normally plays, particularly during development, in calibrating the brain’s representation of auditory space.

Our perceptions of the world are often determined by the way different sensory modalities interact. In the case of hearing, for example, the ability of human listeners to identify sounds such as speech—particularly against a noisy background—or to judge their locations can be profoundly influenced by the availability of concurrent visual cues (reviewed by Alais et al. 2010). It is therefore not surprising that loss of vision can result in changes in auditory perceptual abilities and in the way sounds are processed within the brain. The nature and extent of these changes depends, however, on numerous factors, including the age at onset and the severity and duration of blindness, the aspect of auditory perception that is measured and, almost certainly, on the degree to which visually-deprived subjects have come to depend on their hearing in their everyday lives (King 2014; Lazzouni and Lepore 2014). A range of auditory and other sensory functions can be altered as a result of blindness but because of the particular importance of vision and hearing for spatial perception and navigation, this article will focus primarily on the impact of blindness on sound localization abilities and the underlying neural substrates.

## Visual influences on sound localization in adulthood

Objects in the external world are often both seen and heard, thereby providing multiple cues about their spatial location. Combining the information available to each sensory system can result in a better estimate of object location than would be possible using either system in isolation (Alais et al. 2010). Indeed, sound localization accuracy declines in darkness (Lewald et al. 2000) or if subjects are blindfolded (Tabry et al. 2013). The impact of vision is further illustrated by the demonstration that misaligned visual cues can bias or capture the perceived location of a sound source, which forms the basis for the so-called ventriloquist illusion (Alais et al. 2010).

This visual dominance arises because the retina provides the brain with relatively high-resolution and reliable spatial information about the visual world, whereas sound localization is based on the detection and interpretation of spatial cues that vary in their usefulness with the amplitude and frequency composition of the sound and the region of space in which it needs to be localized (Schnupp et al. 2011). Importantly, however, blurring visual stimuli so that they become harder to localize causes the ventriloquist illusion to work in reverse, with spatially disparate sounds now biasing visual judgments (Alais and Burr 2004). Consequently, rather than vision having an inherent spatial advantage over hearing, it is more appropriate to regard the process by which they are combined in the brain as an example of optimal cue integration, with the weights given to each cue being proportional to the relative reliability of that cue.

Studies of young children suggest that the developing brain may not integrate multisensory signals in the same statistically optimal fashion (Gori et al. 2008, 2012). Instead, it appears that children show strong unisensory dominance, with the dominant sensory modality depending on the task in question, which for visual–auditory space perception is vision (Gori et al. 2012). These authors proposed that the dominant modality calibrates the others during development, with vision therefore contributing to the emergence of auditory spatial abilities. This is consistent with neurophysiological evidence in animals that vision plays the key role in aligning the maps of space for different sensory modalities in the superior colliculus (King 2009). In this case, the developing auditory map is refined by experience so that it becomes aligned with the representation of visual space.

In view of these findings, it might be expected that early blindness would disrupt the normal process of crossmodal calibration, potentially altering the development of sound localization abilities. Indeed, both haptic (Gori et al. 2010) and auditory (Gori et al. 2014) processing deficits have been described in visually impaired individuals. On the other hand, studies in humans (Lazzouni and Lepore 2014) and other species (Rauschecker 1995) have shown that loss of one sensory modality can trigger a functional reorganization, particularly at the level of the cerebral cortex, expanding the neural territory available for processing information provided by the intact senses. Moreover, since blind individuals have to rely more on their hearing for their spatial awareness, it is likely that use-dependent plasticity will sharpen their auditory spatial skills, in much the same way that blind individuals who are adept at reading Braille show heightened tactile spatial acuity that is specific to their reading finger (Wong et al. 2011). These changes therefore have the potential to compensate for the lack of vision.

## Sound localization abilities in the blind

A number of studies have reported that the ability of early blind subjects to localize sound differs from that of sighted controls. To some extent, methodological differences between these studies limit the conclusions that can be drawn but there is now clear evidence that visually impaired humans (Ashmead et al. 1998; Lessard et al. 1998; Röder et al. 1999) and other species (Rauschecker 1995; King and Parsons 1999) can localize sounds in the horizontal plane as well as and often better than sighted individuals. These enhanced auditory spatial abilities are more pronounced for peripheral than for central regions of space (Rauschecker 1995; King and Parsons 1999; Röder et al. 1999; Voss et al. 2004).

Sound localization in the horizontal plane depends principally on binaural spatial cues, i.e., differences between the two ears in sound level and arrival time, which vary systematically in value with the angular direction of the sound source relative to the head. Although experience-dependent plasticity in the processing of binaural cues can occur (Keating and King 2013; Keating et al. 2015), there is evidence that heightened sensitivity to spectral localization cues—which result from the direction-dependent filtering properties of the head and external ears—underpins the superior localization abilities shown by some blind individuals (Doucet et al. 2005; Voss et al. 2011). This is consistent with a greater dependence on monaural spectral cues for localization in the horizontal plane in individuals in which binaural cues are compromised as a result of hearing loss in the contralateral ear (Kumpik et al. 2010; Keating et al. 2013; Agterberg et al. 2014).

Not all studies, however, have reported improved spatial hearing in blind subjects. In contrast to the superior azimuthal performance that is often observed, localization in the midsagittal plane tends to be worse than in sighted controls (Zwiers et al. 2001; Lewald 2002). Because vertical localization is based primarily on spectral cues (Carlile et al. 2005; Tollin et al. 2013), this result seems difficult to reconcile with an improvement in sensitivity to these cues providing the basis for more accurate localization in the horizontal plane. It seems likely that different spectral features are selectively weighted under these conditions but unravelling how this happens will require a better understanding of how spectral shape information is processed in the brain and integrated with binaural inputs. In addition to their impaired elevation judgments, blind subjects appear to struggle with more complex spatial tasks, illustrated by the raised thresholds exhibited relative to normal-sighted controls on a task in which they had to estimate the relative location of the second sound source in a sequence of three sounds presented from different directions in the horizontal plane (Gori et al. 2014). Together, these findings show that only some aspects of spatial hearing improve following blindness, which highlights the importance of the choice of behavioral task when investigating the crossmodal plasticity that results from loss of vision.

## Neural substrates for altered hearing in the blind

**Fig. 1:**
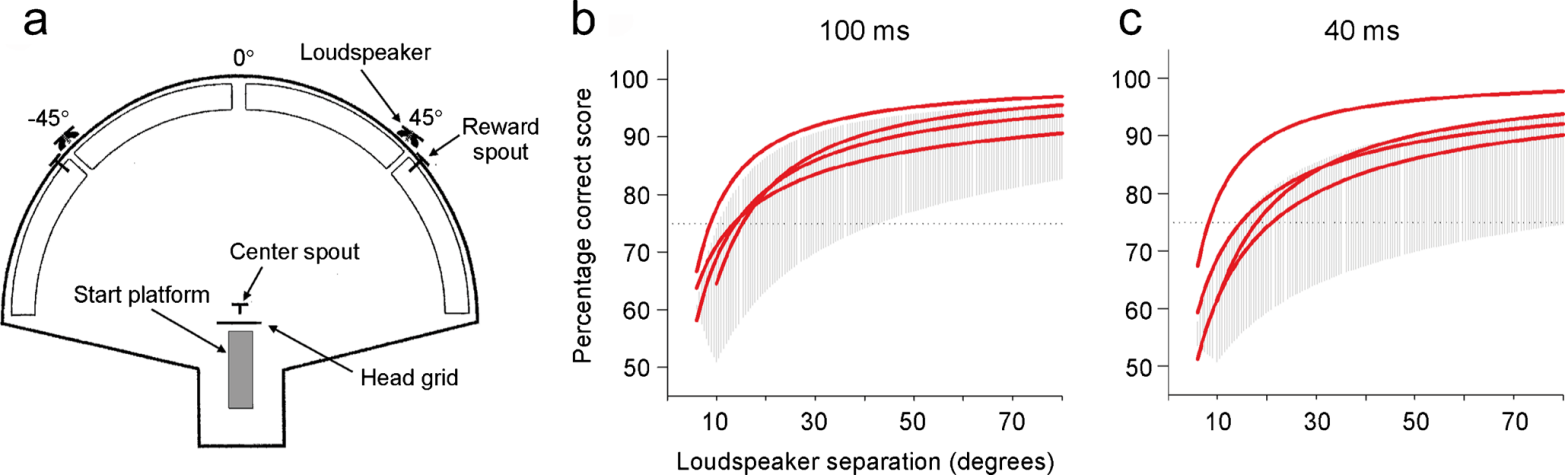
Effects on auditory spatial acuity of depriving ferrets of patterned visual cues during development. **a** Testing arena used to measure the spatial acuity of adult animals at the midline. The ferrets were also tested in the lateral sound field with the loudspeakers placed symmetrically around 45° to one side. A trial was initiated when a ferret stood on the central start platform, placed its head through the head grid and made contact with the center spout. This triggered the presentation of a noise burst, which was selected at random from one of the two loudspeakers. In response, the ferret had to approach and lick the response spout positioned closest to the loudspeaker. **b**, **c** Logistic curves fitted to the psychometric functions in the lateral sound field for 4 visually-deprived ferrets. The shaded region represents the range of data from a normal-sighted control group. The stimuli were either 100 ms (**b**) or 40 ms (**c**) in duration. The performance of the visually-deprived animals was less variable than that of the sighted ferrets and their psychometric functions fell either just above or in the upper range for the control group. No difference was found, however, at the midline. Adapted, with permission, from King and Parsons (1999)

As previously mentioned, visual cues are used to calibrate the developing auditory space map in the superior colliculus (King 2009). Removal of these guiding signals in infancy has been found to impair the maturation of auditory spatial tuning in this midbrain nucleus, with the extent of the changes observed varying with the species and method of visual deprivation used (King and Carlile 1993; Withington-Wray et al. 1990; Wallace et al. 2004). For example, binocular eyelid suture in young ferrets results in normal auditory spatial selectivity in the superior colliculus but an abnormally high proportion of neurons that are ambiguously tuned to two different sound directions (King and Carlile 1993). Lid-sutured ferrets show no impairment, however, in the accuracy with which they approach sound sources in the horizontal plane (Kacelnik et al. 2006), while their spatial acuity in the lateral sound field is as good as and sometimes superior to that of normal-sighted control animals (King and Parsons 1999) (Fig. 1). Complete elimination of visual cues by rearing either guinea pigs (Withington-Wray et al. 1990) or cats (Wallace et al. 2004) in the dark has a more disruptive effect on auditory space map development. Although the sound localization abilities of these animals were not tested, it is likely that the crossmodal plasticity observed in the superior colliculus is more related to the maturation of a capacity for integrating spatial information across the senses than the acquisition of spatial hearing abilities per se.

Surprisingly little work has been done on the effects of visual deprivation on the spatial selectivity of auditory cortical neurons. Korte and Rauschecker (1993) reported that neurons recorded in the anterior auditory cortex of lid-sutured cats show sharper spatial tuning compared to that seen in control animals. Although the anterior auditory field is not thought to contribute to the sound localization abilities of normal cats, the region sampled in this study included the anterior ectosylvian sulcus, which is necessary for determining sound source location (Lomber et al. 2007). Other studies have reported an expansion in the size of the tonotopically organized core auditory cortex in blind human subjects (Elbert et al. 2002), while placing young mice in the dark for a few days leads to sharper frequency selectivity and an improved capacity to discriminate changes in sound frequency and level among neurons in the primary auditory cortex (Petrus et al. 2014). It is possible that such changes contribute to the enhanced pitch discrimination reported in early blind human listeners (Gougoux et al. 2004).

**Fig. 2:**
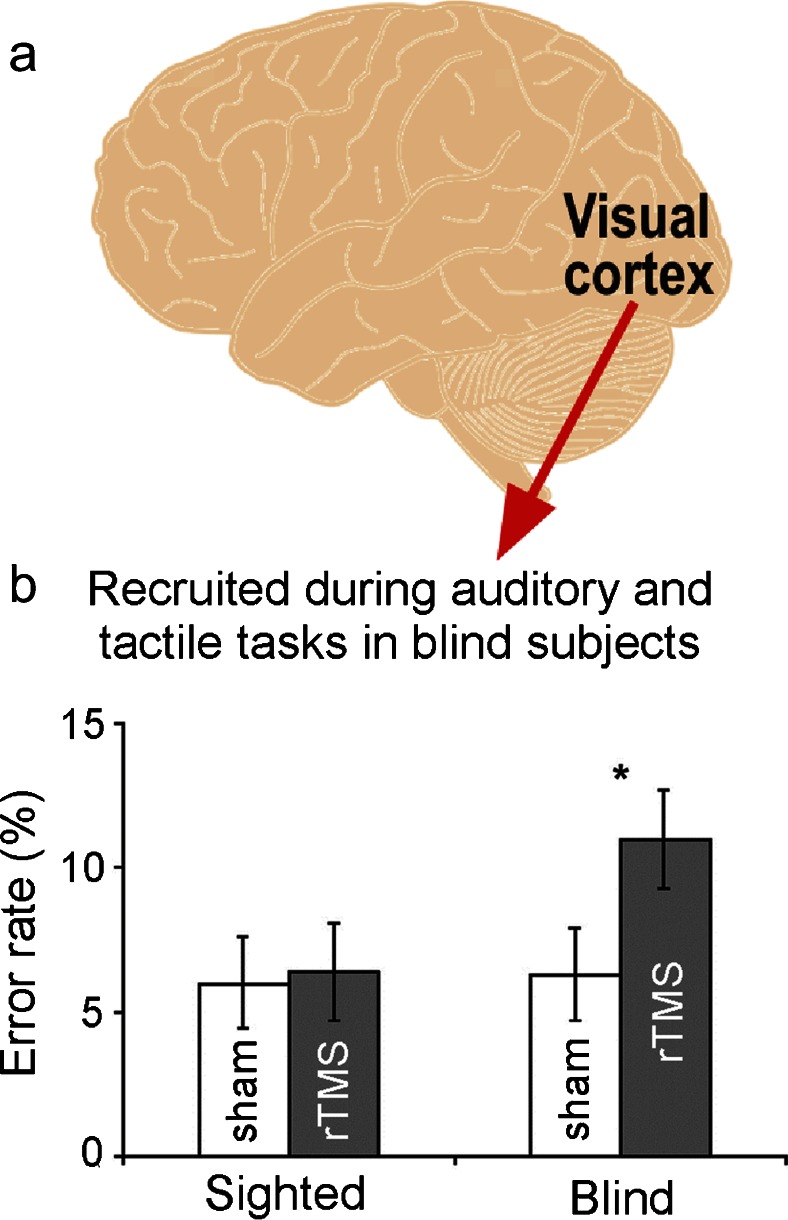
**a** Functional reorganization of the visual cortex in blind subjects contributes to their improved performance in auditory and tactile tasks. In particular, occipital cortex activity correlates with superior performance of blind subjects when they localize sound using spectral shape cues. **b** Transient disruption of the right dorsal extrastriate cortex via rTMS (*gray bars*) produces a significant increase in auditory localization error rate in blind but not in sighted subjects. Adapted, with permission, from Collignon et al. (2007)

Improvements in hearing abilities following blindness may also result from an increase in cortical territory devoted to auditory processing (Rauschecker 1995; Lazzouni and Lepore 2014). In particular, a number of studies have described structural and functional changes within the occipital cortex, with regions that would normally be involved in visual functions now responding to sound. Several lines of evidence suggest that this crossmodal reorganization is behaviorally relevant. First, the extent to which visual cortex is activated in blind subjects correlates with their sound localization accuracy (Gougoux et al. 2005; Voss et al. 2011). Second, functionally appropriate visual cortical areas are recruited following blindness, a principle that underpins the use of sensory substitution devices that convert visual information into auditory signals (Striem-Amit et al. 2012). For example, regions of the occipital cortex activated during auditory spatial processing in early blind individuals are also involved in visual spatial processing tasks in normal-sighted subjects (Renier et al. 2010; Collignon et al. 2011). Third, activation of visual cortical areas can be directly related to perception. Thus, unlike sighted controls, the perceived direction of auditory motion can be accurately classified from activity recorded in the middle temporal complex but not from auditory cortical areas (Jiang et al. 2014). Furthermore, application of repetitive transcranial magnetic stimulation (rTMS) to the right dorsal extrastriate cortex impairs auditory spatial but not pitch or level discrimination in blind subjects, whereas this has no effect on the ability of sighted subjects to perform any of these tasks (Collignon et al. 2007) (Fig. 2).

Although the age at which vision is lost has a bearing on the subsequent crossmodal plasticity in the brain and the way perceptual abilities change (Lazzouni and Lepore 2014), superior sound localization has been observed following both early and late visual deprivation (King and Parsons 1999; Voss et al. 2004; Fieger et al. 2006). Even more striking is the finding that blindfolding normal-sighted adult humans for short periods can result in a transient increase in sound localization accuracy (Lewald 2007). Since a 5-day visual deprivation period can lead to behaviorally-relevant tactile responses in the occipital cortex (Merabet et al. 2008), it is likely that these short-term changes reflect the unmasking of existing connections between brain regions that process inputs from different sensory modalities. In addition, more prolonged deprivation originating early in development probably results in abnormal patterns of connectivity. Indeed, it has been reported that early blind subjects show increased stimulus-dependent coupling between activity in auditory and visual cortical areas (Schepers et al. 2012), as well as greater auditory to visual cortex connectivity compared with sighted controls (Klinge et al. 2010). Other recent evidence suggests, however, that functional connectivity in early blind subjects is actually reduced between visual and auditory cortices but increased between visual cortex and frontal and parietal areas involved in cognitive processing. This suggests that occipital cortex activation during auditory tasks may reflect stronger top–down attentional control in blind subjects (Burton et al. 2014).

## Concluding remarks

A large number of studies have now examined the impact of loss of vision on auditory (and tactile) processing. Because of the greater reliance that visually-deprived humans and animals have to place on their intact sensory modalities, it is not surprising that their behavioral performance is often superior to that of sighted controls. Nevertheless, even for the same function, such as sound localization, conflicting results have been obtained. Although some of this variation can be attributed to methodological differences between studies and to differences in the age at onset, duration and severity of blindness, it is clear that certain aspects of spatial hearing can be enhanced, whereas others are impaired in visually-deprived individuals. This likely reflects a trade-off between the lifelong influence of vision in calibrating neural representations of auditory space and the compensatory crossmodal plasticity that results from a combination of reduced inputs in one sensory modality and increased use of the others. In addition, the opposing effects of blindness on localization based on auditory spectral cues in the horizontal and vertical planes suggest that there may be a limited capacity for experience-dependent improvements in neural processing. Further investigation, particularly at a neuronal level, of the location and nature of the changes that take place following blindness will be key to revealing both the mechanisms underlying crossmodal plasticity and the functional and modality specificity of different brain regions.

## References

[CR1] Agterberg MJ, Hol MK, Van Wanrooij MM, Van Opstal AJ, Snik AF (2014). Single-sided deafness and directional hearing: contribution of spectral cues and high-frequency hearing loss in the hearing ear. Front Neurosci.

[CR2] Alais D, Burr D (2004). The ventriloquist effect results from near-optimal bimodal integration. Curr Biol.

[CR3] Alais D, Newell FN, Mamassian P (2010). Multisensory processing in review: from physiology to behaviour. Seeing Perceiving.

[CR4] Ashmead DH, Wall RS, Ebinger KA, Eaton SB, Snook-Hill MM, Yang X (1998). Spatial hearing in children with visual disabilities. Perception.

[CR5] Burton H, Snyder AZ, Raichle ME (2014). Resting state functional connectivity in early blind humans. Front Syst Neurosci.

[CR6] Carlile S, Martin R, McAnally K (2005). Spectral information in sound localization. Int Rev Neurobiol.

[CR7] Collignon O, Lassonde M, Lepore F, Bastien D, Veraart C (2007). Functional cerebral reorganization for auditory spatial processing and auditory substitution of vision in early blind subjects. Cereb Cortex.

[CR8] Collignon O, Vandewalle G, Voss P, Albouy G, Charbonneau G, Lassonde M, Lepore F (2011). Functional specialization for auditory-spatial processing in the occipital cortex of congenitally blind humans. Proc Natl Acad Sci U S A.

[CR9] Doucet ME, Guillemot JP, Lassonde M, Gagné JP, Leclerc C, Lepore F (2005). Blind subjects process auditory spectral cues more efficiently than sighted people. Exp Brain Res.

[CR10] Elbert T, Sterr A, Rockstroh B, Pantev C, Müller MM, Taub E (2002). Expansion of the tonotopic area in the auditory cortex of the blind. J Neurosci.

[CR11] Fieger A, Röder B, Teder-Sälejärvi W, Hillyard SA, Neville HJ (2006). Auditory spatial tuning in late-onset blindness in humans. J Cogn Neurosci.

[CR12] Gori M, Del Viva M, Sandini G, Burr D (2008). Young children do not integrate visual and haptic form information. Curr Biol.

[CR13] Gori M, Sandini G, Martinoli C, Burr D (2010). Poor haptic orientation discrimination in nonsighted children may reflect disruption of cross-sensory calibration. Curr Biol.

[CR14] Gori M, Sandini G, Burr D (2012). Development of visuo-auditory integration in space and time. Front Integr Neurosci.

[CR15] Gori M, Sandini G, Martinoli C, Burr DC (2014). Impairment of auditory spatial localization in congenitally blind human subjects. Brain.

[CR16] Gougoux F, Lepore F, Lassonde M, Voss P, Zatorre RJ, Belin P (2004). Neuropsychology: pitch discrimination in the early blind. Nature.

[CR17] Gougoux F, Zatorre RJ, Lassonde M, Voss P, Lepore F (2005). A functional neuroimaging study of sound localization: visual cortex activity predicts performance in early-blind individuals. PLoS Biol.

[CR18] Jiang F, Stecker GC, Fine I (2014). Auditory motion processing after early blindness. J Vis.

[CR19] Kacelnik O, Nodal FR, Parsons CH, King AJ (2006). Training-induced plasticity of auditory localization in adult mammals. PLoS Biol.

[CR20] Keating P, King AJ (2013). Developmental plasticity of spatial hearing following asymmetric hearing loss: context-dependent cue integration and its clinical implications. Front Syst Neurosci.

[CR21] Keating P, Dahmen JC, King AJ (2013). Context-specific reweighting of auditory spatial cues following altered experience during development. Curr Biol.

[CR22] Keating P, Dahmen JC, King AJ (2015). Complementary adaptive processes contribute to the developmental plasticity of spatial hearing. Nat Neurosci.

[CR23] King AJ (2009). Visual influences on auditory spatial learning. Philos Trans R Soc Lond B.

[CR24] King AJ (2014). What happens to your hearing if you are born blind?. Brain.

[CR25] King AJ, Carlile S (1993). Changes induced in the representation of auditory space in the superior colliculus by rearing ferrets with binocular eyelid suture. Exp Brain Res.

[CR26] King AJ, Parsons CH (1999). Improved auditory spatial acuity in visually deprived ferrets. Eur J Neurosci.

[CR27] Klinge C, Eippert F, Röder B, Büchel C (2010). Corticocortical connections mediate primary visual cortex responses to auditory stimulation in the blind. J Neurosci.

[CR28] Korte M, Rauschecker JP (1993). Auditory spatial tuning of cortical neurons is sharpened in cats with early blindness. J Neurophysiol.

[CR29] Kumpik DP, Kacelnik O, King AJ (2010). Adaptive reweighting of auditory localization cues in response to chronic unilateral earplugging in humans. J Neurosci.

[CR30] Lazzouni L, Lepore F (2014). Compensatory plasticity: time matters. Front Hum Neurosci.

[CR31] Lessard N, Paré M, Lepore F, Lassonde M (1998). Early-blind human subjects localize sound sources better than sighted subjects. Nature.

[CR32] Lewald J (2002). Vertical sound localization in blind humans. Neuropsychologia.

[CR33] Lewald J (2007). More accurate sound localization induced by short-term light deprivation. Neuropsychologia.

[CR34] Lewald J, Dörrscheidt GJ, Ehrenstein WH (2000). Sound localization with eccentric head position. Behav Brain Res.

[CR35] Lomber SG, Malhotra S, Hall AJ (2007). Functional specialization in non-primary auditory cortex of the cat: areal and laminar contributions to sound localization. Hear Res.

[CR36] Merabet LB, Hamilton R, Schlaug G, Swisher JD, Kiriakopoulos ET, Pitskel NB, Kauffman T, Pascual-Leone A (2008). Rapid and reversible recruitment of early visual cortex for touch. PLoS ONE.

[CR37] Petrus E, Isaiah A, Jones AP, Li D, Wang H, Lee HK, Kanold PO (2014). Crossmodal induction of thalamocortical potentiation leads to enhanced information processing in the auditory cortex. Neuron.

[CR38] Rauschecker JP (1995). Compensatory plasticity and sensory substitution in the cerebral cortex. Trends Neurosci.

[CR39] Renier LA, Anurova I, De Volder AG, Carlson S, VanMeter J, Rauschecker JP (2010). Preserved functional specialization for spatial processing in the middle occipital gyrus of the early blind. Neuron.

[CR40] Röder B, Teder-Sälejärvi W, Sterr A, Rösler F, Hillyard SA, Neville HJ (1999). Improved auditory spatial tuning in blind humans. Nature.

[CR41] Schepers IM, Hipp JF, Schneider TR, Röder B, Engel AK (2012). Functionally specific oscillatory activity correlates between visual and auditory cortex in the blind. Brain.

[CR42] Schnupp J, Nelken I, King A (2011). Auditory neuroscience: making sense of sound.

[CR43] Striem-Amit E, Dakwar O, Reich L, Amedi A (2012). The large-scale organization of "visual" streams emerges without visual experience. Cereb Cortex.

[CR44] Tabry V, Zatorre RJ, Voss P (2013). The influence of vision on sound localization abilities in both the horizontal and vertical planes. Front Psychol.

[CR45] Tollin DJ, Ruhland JL, Yin TC (2013). The role of spectral composition of sounds on the localization of sound sources by cats. J Neurophysiol.

[CR46] Voss P, Gougoux F, Lassonde M, Fortin M, Guillemot JP, Lepore F (2004). Early- and late-onset blind individuals show supra-normal auditory abilities in far space. Curr Biol.

[CR47] Voss P, Lepore F, Gougoux F, Zatorre RJ (2011). Relevance of spectral cues for auditory spatial processing in the occipital cortex of the blind. Front Psychol.

[CR48] Wallace MT, Perrault TJ, Hairston WD, Stein BE (2004). Visual experience is necessary for the development of multisensory integration. J Neurosci.

[CR49] Withington-Wray DJ, Binns KE, Keating MJ (1990). The maturation of the superior collicular map of auditory space in the guinea pig is disrupted by developmental visual deprivation. Eur J Neurosci.

[CR50] Wong M, Gnanakumaran V, Goldreich D (2011). Tactile spatial acuity enhancement in blindness: evidence for experience-dependent mechanisms. J Neurosci.

[CR51] Zwiers MP, Van Opstal AJ, Cruysberg JR (2001). Two-dimensional sound-localization behavior of early-blind humans. Exp Brain Res.

